# The Impact of Financial Hardship on Single Parents: An Exploration of the Journey From Social Distress to Seeking Help

**DOI:** 10.1007/s10834-017-9551-6

**Published:** 2017-10-17

**Authors:** Rebecca Jayne Stack, Alex Meredith

**Affiliations:** Department of Psychology, School of Social Sciences, Nottingham Trent University, Nottingham, UK

**Keywords:** Financial hardship, Help-seeking, Psychological distress, Single parents

## Abstract

Single parent families are at high risk of financial hardship which may impact on psychological wellbeing. This study explored the impact of financial hardship on wellbeing on 15 single parents. Semi-structured interviews were conducted and analysed using constructivist thematic analysis. Participants described food and fuel poverty, and the need to make sacrifices to ensure that children’s basic needs were met. In some cases, participants went without food and struggled to pay bills. Isolation, anxiety, depression, paranoia, and suicidal thoughts were described. However, participants reported that psychological services not able to take the needs of single parents in to account. Support for single parents must acknowledge the impact of social circumstances and give more consideration economic drivers of distress.

## Background

In the United Kingdom, approximately one in four children live in single parent families (also known as lone parent families). In 2016 there were 2.9 million single parents in the United Kingdom, representing an 18.6% increase in single parents since 1996, (Great Britain. Office for National Statistics 2016). Women account for 86% of single parents with dependent children, the average age of a single parent is 38 years of age, with approximately 60% of single parents caring for one dependent child. Single parent families are one representation of the range and diversity of family units in modern society (Golombok 2000; Golombok et al. 2016) and can be created through circumstances, including divorce, separation, death of a partner, donor insemination or an unplanned pregnancy.

Societal perceptions often construct single parents as young, female, unemployed parents with multiple children (Garner and Paterson 2014; Zartler 2014). Single parents are a stigmatised group in that they are in possession of a set of characteristics that conveys a social identity that is often devalued within society (Crocker et al. 1998). However, in Britain, employment among female single parents is higher than that of married or co-habiting women (Chambaz 2001). Despite high employment levels single parents are more likely to experience fuel poverty than other family structures (Liddell 2008). In addition, single parent families are still nearly twice as likely to be in poverty as those in couple parent families, with 67% of single parents reporting that they struggle with finances (Gingerbread 2015). Single parents therefore must manage a number of stressors including stigma, work and poverty.

The link between financial hardship poor health and poor mental health has been demonstrated in multiple populations. A study across 27 European countries found that single parents (in comparison to cohabiting parents and married parents) had poorer health, with the United Kingdom being substantially worse in this regard (Campbell et al. 2015; Van de Velde et al. 2014). In addition, studies have shown that single parents also experience lower levels of mental health and low psychological wellbeing (Ifcher and Zarghamee 2014), with more extensive use of the mental health services (Cairney and Wade 2002). Brown and Morgan (1997) examined marital status, poverty and depression in female parents over a 2-year period and found that single parents were twice as likely as their married counterparts to be in financial hardship (Brown and Moran 1997), despite being twice as likely to be in full-time employment. Single parents have been shown to experience higher levels of chronic stress (Cairney et al. 2003), loneliness (Baranowska-Rataj et al. 2014) and depression (Jackson et al. 2000). Elevated distress levels were also identified in German single parents compared to married mothers (Franz et al. 2003). Tein et al. (2000) conducted a prospective longitudinal study of the relationships among life stress, psychological distress, coping, and parenting behaviours in single mothers in the United States. The findings showed that both major and minor events had a significant impact on distress levels, with daily negative events having the largest impact on distress levels. Theoretically, high levels of distress, low economic resources and a lack of stress buffering resources may lead to poor psychological coping strategies amongst single parents (Folkman and Lazarus 1980), however, this must be explored though the in-depth examination of single parent experience.

It is clear from existing research that single parents are likely to experience higher levels of depression, anxiety, and general stress, despite making extensive efforts to meet their financial obligations. However, there is little research exploring help-seeking and how distress relating to financial hardship is addressed. The aims of this study were to explore the impact of financial hardship on personal health and wellbeing on single parents, and their attempts to seek help to cope with the impact of financial hardship.

## Methods

### Participants

Participants were recruited from single parent networks and charities in England, such as Gingerbread (a British charity offering advice, support and networking opportunities to single parents), with the recruitment being enabled through posters, flyers, and social media announcements. Eligible participants were parents who were currently raising at least one dependent child in a household consisting of one adult. In addition, the participants of this study self-identified with the terms lone parent or single parent. Demographic details and the parenting responsibilities of participants are presented in Table 1.

**Table 1:** Characteristics of participants

Sabrina	47	One dependant (age 15)	Single parent for 15 years	Working full time
Sheryl	36	One dependant (age 9)	Single parent for 5 years	Working part-time
Emily	28	One dependant (age 3)	Single parent for 3 years	Working part-time
Laura	45	Two dependants (age 8 and 15)	Single parent for 6 years	Working full-time
Alexandria	36	One dependant (aged 6)	Single parent for 6 years	Working full-time
Sarah	33	One dependant (age 9)	Single parent for 7 years	Stopped work due to illness
Zainab	34	One dependant (age 7)	Single parent for 1 year	Returning to work following extended leave
Charlotte	55	One dependant (age 16)	Single parent for 22 years	Retired, and undertaking voluntary work
Alisha	29	One dependant (age 2)	Single parent for 3 years	Working part-time
Stella	35	Two dependents (age 5 and 4)	Single parent for 6 years	Working full-time
Claire	37	Two dependents (age 3 and 5)	Single parent for 2 years	Working full-time
Martha	40	Two dependents (aged and 5)	Single parent for 1 year	Self-employed
Sandra	23	One dependant (age 3)	Single parent for 2 years	Recently made redundant
Sara	25	One dependant (age 9 months)	Single parent for 1 year	Returning to work following child-birth
Rosemary	32	Two dependants (age 5 and 12)	Single parent for 4 years	Working full-time

### Procedure

Ethical approval for this procedure was granted by a University Ethics committee. The first author conducted 15 one-to-one semi-structured interviews which were guided by an interview schedule. The interview schedule was developed through consultation with single parent charities and a literature review on financial hardship, wellbeing and parenting. Following the first two interviews, the schedule was adapted to reflect the emerging topics and themes discussed by interviewees, with existing questions also being adjusted as appropriate. This inductive adaption of the interview schedule was undertaken again after a further four interviews had been conducted. Interviews lasted between 30 to 90 min, were audio-recorded using a digital voice recorder and transcribed verbatim prior to analysis.

The data were analysed using thematic analysis (Braun and Clarke 2006), with a constructivist epistemology being applied to the interpretation of the emerging themes. The initial stage of analysis was line-by-line coding where labels were attached to descriptions, events, perceptions and topic of discussion featured in the interview transcript. The first five interview transcripts were independently coded by the first and second authors. Coding categories that lacked concordance were discussed and absorbed into the coding framework. The initial codes were then grouped into the most noteworthy and frequently occurring global themes. The quotations within each theme were arranged into organising themes with constructivist descriptions of quotations developed by the first author, the constructivist descriptions were drawn from a combination of the quotations content, the line-by-line codes, and the relationship between the quotations within the organising themes. In addition, relationships between the organising themes were considered, and their overall contribution towards their global theme were outlined.

## Findings

All participants were female between 18 and 55 years of age. The findings are organised in to core themes, related to the impact of financial hardship, debt on psychological wellbeing and health (Table 2).

**Table 2:** Table of organising and sub-themes

Organising theme	Sub-themes
The stress of being a single provider	Lone responsibility Making sacrifices
The impact of financial hardship	Physical Health Mental Health
Seeking help and support	Foodbanks Mental Health Support

### The Stress of Being a Single Provider

#### Lone Financial Responsibility

The parent who took the primary custodial responsibility for the child(ren) was construed to be the parent with the primary financial burden; a scenario which was described by one participant as *big trouble*. Not having enough financial resources to support their children and needing to rely on other people were described as stressful and worrying. Participants represented themselves as trapped and helpless, and constructed their position as one where there was a persistent battle with finances; with the need to *fight for everything*. Participants described a range of feelings in response to the constant worry about providing for a child a single person, including feeling miserable and stressed. These feelings and stressors did not dissipate with time, and were worsened by unanticipated life events.

I’m miserable really. It’s, it’s stress. It’s the stress of it. It’s, it’s worrying every single day how, how you’re gonna give your child the best…I’m in this position with a child I feel completely helpless. I can’t support my own family and I have to rely on what I’m given and you have to fight everything nowadays. (Emily, a 28 year old mother of one young child)

Participants viewed money worries as being a central part of everyday life, and was a repeated stressor which could not be avoided. The stress and the worry about food was constructed as being constant and life-consuming, and was therefore something that regularly occupied their thoughts. It was this sense of rumination about being unable to provide necessities that lead to feelings of sickness, with some single parents describing the bleakness of their current circumstances, with feelings of hopelessness and depression. Often at the centre of this worry were concerns about providing food, which were constructed as part of an ongoing daily struggle. These food related worries were linked by participants to sleeplessness, and feelings of physical sickness (described in more detail in Theme 2).

I really need to do some food shopping but I’ve got £4…I’ve had sleepless nights and nights full of tears, where I’ve just thought I literally don’t know how I’m going to get through the next few days. I’ve got no food, no money…So yeah, definitely times where I’ve felt very, very depressed about the situation and can’t see a way out of it almost. (Sandra, a 23 year old mother of one young child)

#### Making Sacrifices

Participants constructed their personal responsibility for feeding children as vital, and unquestionable. Participants described doing whatever it took to ensure that their children had food, so that their children would not go hungry. In doing so, however, participants would reduce their own food intake or *go hungry*, arguably leading to the detriment of their own physical and mental health. Similarly, meals would be skipped to pay utility bills, or to ensure that debt associated with bills was not accumulated.

I don’t eat sometimes; I just have my online shopping and it’s all for my daughter, so I’ll be having toast for dinner. That’s, that’s kind of life really. (Alisha, a 29 year old mother of one infant child)

Um, there are cases where I will skip meals. Um, you know, there was a few weeks where it was literally like “Right, I’ve got £5 at the end of the week or whatever, this has either got to go on electric or, or something else” and I will skip meals. (Sarah, a 33 year old mother of one young child)

Financial hardship was associated with household fuel poverty, with some participants specifically speaking about the difficulties they faced in providing heat in their homes (as opposed to other forms of fuel consumption). Participants discussed making decisions not to heat their homes to save money, or lowering the temperature of heaters to reduce financial outgoings. Fuel costs were presented as an acceptable necessity to sacrifice, where as providing food was never questioned. In a similar way to identifying funds for food and heating, finding the money for children’s clothing was a source of worry. School clothing was suggested to be a particular burden, which could not be avoided, requiring participants to worry about *finding the money*.

But there were times when I thought you know, I’m not going to put my gas on. And I’m not going to do that extra load of washing, just because I don’t know if I can afford it, and I need to make sure I’ve got money in my purse to go and do food shopping. (Sandra, a 23 year old mother of one younger child)

He’s starting secondary school in September and I’m going to have to find the money for all his school uniform and blazers and that’s gonna run in to hundreds I dare say. So I’m already thinking “oh my goodness, how am I gonna find the money for that?” (Sarah, a 33 year old mother of one young child)

Single parent participants also suggested that financial hardship had a negative impact on their social interactions. Social isolation, loneliness and withdrawal were suggested to corrode their psychological wellbeing and mental health. Not having enough money to participate in social activities was suggested to be a physical barrier, however, the embarrassment of having little money was a social and psychological barrier which was suggested to underlie their tendency to withdraw socially. This meant that social withdrawal and social isolation were often associated with financial stress and financial hardship.

And I think I’d love to be able to take him to a Soft Play, or do something else that normal parents would be able to do, but I can’t because I haven’t got any money. (Martha, a 40 year old mother of two young children)

But it’s just little things, like if we decide to go to the park with a friend, they might get a treat or go for a coffee. And I just thought I don’t have three pounds to do that, so we’ll just stay at home and do things ourselves. (Sabrina, a 47 year old mother of a teenager)

### The Impact of Financial Hardship

#### Physical Health

Participants often questioned their ability to *cope*. In some cases participants described feeling so ill that they were unable to eat, with the stress related to work and the need to bring in extra money leading to exhaustion. A large proportion of physical symptoms were related to strain, stress, and feeling run down which were described in ways that directly related these physical symptoms to psychological wellbeing. For example, the accumulative effect of stress related to money worries and sole responsibility were suggested to interrupt sleep and lead to sleeplessness. The psychological impact of night-time excessive rumination experienced by single parents was inescapable, but in some cases participants forced themselves to get physical rest. However, despite forcing physical rest, some participants described feeling *run down*. The stress of doing *everything* was linked to more susceptible to illness, particularly colds and flu.

Yeah, oh I felt so ill, I couldn’t eat, it would just come over in waves, it was awful…I was doing a bit of cleaning as well to bring in some money to pay the food and I think the body just, said I can’t cope. (Charlotte, a 55 year old mother of a teenager)

I didn’t really cope very well, I just, I used to have sleepless nights, if I woke up I would often go and I would, things would go round and round and round in my head all the time, erm, so at night I would maybe get 3 h sleep…You’re doing everything, so yes it does, it puts that extra strain on you. So I would definitely say yes, and you’re picking up more, because you’re run down all the time, I think you’re more likely to pick up bugs and things as well. (Laura, a 45 year old mother of one young child and one teenager)

Participants described how they would cope with illnesses such as chest infections and back pain that caused them to seek medical attention, but highlighted that they were unable to rest and recover. Regardless of one’s health status or the presence of an illness the need to do *everything* remained. Some participants, such as Zainab, also suffered from long-term illnesses, which presented an extra physical challenge. Here the need for rest was particularly pertinent, and a range of strategies designed to juggle illness alongside their parenting responsibilities were described. Often, normal sick role activities (such as rest and seeking help) were inhibited by the responsibilities of parenting alone.

I just have to sleep and rest when I can, when I haven’t got him or when he’s at school. Um, I have to try and pace myself in terms of trying to get the housework done. (Zainab, a 34 year old mother of one young child)

If you’re ill, there was no way I would get to lie in. So I did find that I was ill quite a lot and I found myself going to the Doctors for chest infections or my back being out because I do suffer with back pain. (Laura, a 45 year old mother of one young child and one teenager)

#### Mental Health

Sleepless nights, frustration and distress were common. Participants described feeling anxious about *everything*, suggesting that single parents were on high alert. Some single parent participants described feeling judged by others and had feelings of paranoia. Similar to physical health, descriptions of mental health were underlined by stress, rumination, and the turmoil of circumstances. Descriptions of feeling overwhelmed constructed the enormity of the situation, and placed the experience of distress as paramount in their existence. Claire also described the exhaustion she felt, and collapsing once the children had gone to bed. All of her energy had been devoted to caring for her children, and once they were asleep, she would spend the time alone in a state of distress which involved exhaustion, crying, feeling overwhelmed, and sleeplessness.

I was so anxious about everything…I felt so judged by everybody. Yeah, VERY anxious… there was a spell when basically the boys went to bed about 7:15, 7:30, and then I just collapsed sobbing on the sofa and then went to bed. And I couldn’t sleep because my head was just in turmoil about all the things that had happened. It just can feel really overwhelming sometimes. (Claire, a 37 year old mother to two young children)

So my situation escalated really quickly…And I just found that with the pressure of looking after two such young children and their care needs, plus the pressures both financially and, you know, sorting out all the bills, having to get things like tax credits which I’ve never had before in my life. Having to look after the house and do all that, and the garden, and the car, and everything by myself, I just kind of imploded. (Sara, a 25 year old mother of an infant child)

The distress and anxiety experienced were linked by participants to parenting responsibilities and financial hardship. In some cases participants were very specific in outlining the source of their distress, for example sorting out bills, tax-credits, taking care of the house and caring for the children. Feelings of entrapment, desperation and being unable to get out of the current situation led to suicidal thoughts. In addition, it was felt that there was not help *out there* for the mental health problems being faced, with Charlotte (a 55 year old mother of a teenager) saying that there was no help to empower people to *steer out of it*. This would indicate that a therapeutic paradigm with more direction would be of benefit. Conversely the presence of their children was protective for their mental health.

I would have killed myself, I know, because I thought about it many times, so that is…it’s desperate, when you’re in a mess, it’s desperate…but I think so much more needs to be done in educating ordinary people in how to manage finance. (Charlotte, a 55 year old mother of a teenager)

He [the child] is the best thing that has ever happened to me in my life. I honestly would have been dead by now if it weren’t for him because I would have just killed myself, like literally. (Alexandria, a 36 year old mother of a young child)

### Seeking Help and Support

#### Seeking Help: Food Banks

Food banks were used as support in times of crisis when they were unable to provide adequate levels of food. Some participants were not aware of the help available at food banks, and were not sure if they were entitled to support. In some cases participant were advised by workers at a SureStart centre (a government run local/community based centre to advice and support to families with young children) that they were entitled, highlighting the vital role of community support in signposting support. Participants presented that this type of identification of needs and signposting was necessary for them to access support, as Martha (a 40 year old mother of two younger children) sated that she  “wouldn't have dreamt of asking for it.”

There’s a difference between knowing it exists. It’s probably one of these things, like a lot of people say about benefits, oh I’m not entitled to it, there’s probably a lot of people what think. (Rosemary, a 32 year old mother of one young child and one teenager)

I was at one of the SureStart Centres one day helping at something, and one of the staff who knew my situation came up and asked how was I getting on…And I said I’m starting to struggle again, and she said well, you do know you can use food bank. I wouldn’t have dreamt of asking for it. I didn’t know that… I knew all about it in a sense, but I didn’t know I was entitled to have it. (Martha, a 40 year old mother of two young children)

For others, the issue was in getting access to the food bank. One single parent described a desperate situation where she was unable to provide food for her daughter, but did not have the money or resources to get to the food bank. In these circumstances the impact of not being able to afford transport, prevented access to services designed for those unable to afford food; with one form of hardship, impacting upon another form of hardship, constructing a cycle of deprivation.

My daughter was eating peanuts and I thought…there’s no way she’s gonna have just peanuts today, and I’d called the council to say, you know, could you help me with the food bank…please, there must be something you can do to help me. I can’t, I’ve got no money to get there. And the lady said “well, sorry, you know, if you can’t get there you can’t have the food.” So even simple things like that, you know, you, you can’t get to a food bank and they’re refusing to kind of help you, you’d think they might be able to bring it to you if you’re, you know, in a really dire situation. (Emily, a 28 year old mother of one young child)

#### Seeking Help: Mental Health Support

Professional help was often sought when participants described themselves as having hit a crisis point. This was usually characterised by the accumulated stress of being a single parent (described in Themes 1 and 2) become overwhelming, and the ability to continue with parenting duties were questioned. The feeling of crisis was constructed as extreme, and at a point where the only option was to seek help. The was an underlying sentiment that help for single parents was not typical or expected, therefore, seeking help was an extraordinary act born out of crisis. Typically, general practitioners (GPs also known as family practitioners) acted as the first line of support in such cases. GPs were constructed as highly responsive, supportive and caring. They often offered antidepressants as intervention, but it appeared that their initial response (of caring and taking the situation seriously) were well received and helpful.

And my GP was brilliant, and he was very supportive. He straight away went down the route of yes, if at a later date we feel the need for anti-depressants and things, but at this precise minute I want to see you every week. Let’s just, you know, keep things talking. (Martha, a 40 year old mother of two young children)

And I went to see my GP, and she was lovely. And I’ve never had any sort of mental health difficulties ever in the past and she just said I think you need some chemical help. (Claire, a 37 year old mother of two young children)

Drug therapies were not always seen as being a viable treatment or as an alternative to psychotherapy. Similarly, participants believed that their distress was caused by social stressors, and that a chemical solution acting on the brain would not address the cause of their distress. The causal beliefs about the origins of their distress (e.g., financial and social), were preventing single parent participants from accepting the feasibility of an intervention. There was a resistance to being *medicated* or reduced to a fluffy state, and that pharmacotherapy would just mask the issue, and that the underlying issues would remain, and would continue to be unaddressed and perhaps worsen. Additionally, one participant was worried that taking antidepressant would make her susceptible to being taken advantage of, or not being fully coherent enough to manage the multiple tasks she was required to undertake. However, there was a suggestion that feelings of anger, depression and sadness were normal and justified, and that interventions attempted to remove these justified feelings, and that help should have an alternative focus which allows single parents to work with these feelings and address the social consequences of their psychological distress.

I didn’t actually take any anti-depressants because my philosophy was, I know what’s causing my depression, erm, and if I could resolve those issues, then my depression would go away, so I didn’t feel that I wanted to mask things. (Laura, a 45 year old mother of one younger child)

If I did take medication, I would become like even more relaxed and maybe even a bit more compliant but you know, I don’t know, I just didn’t want any of my sense to be reduced, I wanted to feel that anger, I had a right to that anger. (Sara, a 25 year old mother of one younger child)

There was a general scepticism to psychological interventions from some participants. One parent described that she felt as though she were just *going over* things. Whereas others suggested that they felt that their psychological state was not the result of disorder thinking, or other traditional causes of mental health difficulties. Instead participants saw themselves as under extreme stress, therefore, their thoughts and feelings were legitimate and did not need to be changed through psychological intervention. There was a desire for more solutions to emerge from the counselling process.

I had six sessions with a counsellor. I’m not sure the counselling helped, to be truthful. It kind of felt like just going over the same stuff, and the counsellor didn’t kind of suggest anything, or say anything. It was just kind of you oh gosh, that’s difficult, but didn’t kind of have any solutions. (Claire, a 37 year old mother of two young children)

Low mood, anxiety and depression were thought by participants to be the result of their social circumstances, therefore, attempts made to change their way of thinking would not address the underlying social, financial and stress origins of their psychological morbidity. Participants described how services were not designed to deal with their complex social needs. Instead services were set up for psychological disorders, but not for psychological disorders where the stressors were external (e.g., parenting responsibility, financial or poverty related). Therefore, participants held scepticism about how effective traditional psychological therapies would be in helping to alleviate their anxiety, depression, distress or suicidal thoughts.

He (GP) then referred me to, what I was told was counselling, but ended up being CBT, cognitive behavioural therapy. And it was a complete waste of time. Because to me, CBT is good if you want to change habits and things. However the stresses and things I was feeling, was not due to any habits that needed changing. It was due to, you know, my life being completely turned upside down. (Martha, a 40 year old mother of two young children)

If you kind of turned up to a healthcare professional and started talking about some of the issues you go through, they don’t actually have services designed to actually help single parents cope or even just a basic understanding of some of the things you’ve been through. So when you kind of rock up to CBT, they’ve got no idea or conceptualisation of what you’ve been through but if you have an eating disorder… (Sheryl, a 36 year old mother of one young child)

## Discussion

This exploration of financial hardship in single parents identified multiple aspects of poverty. Single parents in this study described making difficult compromises to afford food, heating, and clothing and often focused their resources on their children. Participants described attempting to shield their children from poverty through missing their own meals, and taking on extra work which meant that the experiential impact of financial stressors were contained by the parent. Many poverty theories conceptualise negative traits in poverty related decision making including impertinence and impulsivity which are thought to perpetuate poverty cycles, this research highlights that parents make decisions which minimise the impact of the on their children, however, such decision make increase the impact on them as individuals. Psychological research has identified that parental self-sacrifice is a negative core belief, which is associated with negative outcomes and often accompany feelings of shame and a lack of control (Shah and Waller 2000). The impact and consequences of self-sacrifice styles of decisions making on factors such as wellbeing and long-term financial hardship should play a role on poverty related decision-making models. Similarly, Bahr and Bahr (2001) noted that self-sacrifice is a concept neglected in family theory, yet self-sacrifice plays a clear role in family dynamics.

Single parents in our sample described high levels of stress, psychological distress and anxiety, which were related to their position of sole responsibility and concerns about finances. Some levels of distress were particularly concerning, including rumination, sleeplessness, and suicidal thoughts. A particular concern, and one which should be addressed by healthcare professionals and policy makers, was that single parents in this study suggested that mental health distress was normal, and that psychological difficulties were a natural consequence of their social circumstances. This finding is concerning as it indicates that single parents are less likely to seek help or support for mental health difficulties. Furthermore, when help was sought the causal beliefs associated with their mental distress affected their ability to engage with psychological interventions. In addition, stress related health concerns were also described, however, many parents the option to rest and recover was removed due to parental duties.

Self-regulation models of help-seeking behaviour examine the role of beliefs in determining whether help is sought for physical or mental health condition (Bishop and Converse 1986). This theory suggests that we hold prototypical beliefs about what it is like to experience an illness, and when we experience symptoms comparisons are made between the expected experience and the actual experience (Jones 1990). Beliefs where comparisons are made between expectations and experience include the cause of an illness, the timeline associated with an illness (how long with it last), the consequences, identity (symptoms associated with the illness) and the ability for the illness to be controlled (Stack et al. 2013). This study identified a range that single parents held prototypical beliefs about mental health conditions, but did not always recognise the legitimacy of their distress, instead suggesting that the cause of their symptoms was normal for people in their social circumstances. This causal belief was a barrier to help-being sought. Further exploration of the prototypical beliefs held by single parents experiencing mental health difficulties is required to understand the role negative self-beliefs and self-stigma on single parents’ willingness to access mental health services. Previous research has explored the role of social interactions in the development of prototypical beliefs (Tiwana et al. 2015), however, this study highlights that other factors such as self-stigma, social stigma and family dynamics can play an important role in the development of prototypical beliefs, and may prevent help-seeking. Developments of self-regulation prototypical models may consider the role of social factors in belief development, and ways that health services may help potential service users to understand that their illness is deserving of treatment.

The occurrence of psychological distress, stress, health concerns originated from stressors were also found in other studies (Campbell et al. 2015; Van de Velde et al. 2014). However, the current study identified the barriers and facilitators of seeking help, particularly, medical attention for the impact of distress, stressors, financial hardship and isolation. Meyer (2003) offered a conceptual framework to describe the impact of stigma, prejudice and discrimination faced by selected social groups. These social factors mean that some social groups face a negative social environment which contributes towards poor mental health. Meyer’s model considered the impact of hostile social environments for Lesbian, gay and bisexual people, but considered how other minority groups are exposed to excess social stressors which increased the prevalence of poor mental health for stigmatised social groups. Single parents are another group potentially effected by minority social stress, as single parents in this study discussed social isolation, social withdrawal and poor mental health. Further research, should explore the impact of stressors within negative social environments and the impact these factors have on mental health, and the ability of stigmatised minority groups in seeking support for mental health conditions in hostile social environments.

Previous research has identified that healthcare professionals routinely identify fuel poverty as a causal factor for numerous health problems, including asthma, and cardiovascular diseases (Atsalis et al. 2016). However, our study highlights that the impact of fuel and food poverty go beyond physical health manifestations and it is essential for healthcare professionals to address the social and psychological consequences of financial concerns and the social stressors associated with being a single parent. This study identified that initial contact with GPs were on the whole useful and positive. However, the further support in the form of counselling, psychotherapy and pharmacotherapy was suggested to be unhelpful. In this study, single parents reported being offered anti-depressants, cognitive behaviour therapy and more traditional forms of counselling. However, approaches which place the experience of social and financial hardship at the forefront of intervention are required. We identified that single parents believed their distress originated in social circumstances, therefore, therapies directed towards addressing cognitions and emotions were rejected. Therapeutic approaches to therapy which considers the social processes relevant to single parents must be promoted and made more readily available. Indeed, in the recent past, approaches to counselling designed to address the needs of single parents have been developed which could prove promising (Atwood and Genovese 2014; Morawetz and Walker 2014). Further research is needed to explore whether access to these therapies can be broadened, and whether more consideration can be given to social processes in healthcare.

This paper highlights a number of number of issues affecting single parents which are important for policy makers and healthcare professionals to consider. Despite financial hardship single parents were unwilling to compromise on food, with this being one area where some single parents, when signposted, sought help. One parent in particular was assisted by staff at a SureStart centre [providers of advice and support for families, covering a range of issues including job opportunities, support with health, social support and child welfare (Glass 1999)]. While some have questioned whether SureStart centres are accessed by disadvantaged and hard-to-reach communities (Hutchings et al. 2007), our study suggests that these centres may be utilised by single parents for advice and signposting. However, recently, funding to these centres has been significantly reduced in 2015, 156 SureStart centres closed, up from the 85 centres closed in 2014 (leaving a total of 3259 centres remaining in 2016 according to the Department of Education). Therefore, this potential source of support is being diminished, and may have an impact upon the welfare and wellbeing of single parent families experiencing poverty (Melhuish et al. 2008).

This qualitative study has explored a number of important issues in a small sample of single parents and this paper is limited in its generalisability to wider single parent populations, however, the findings have formulated the basis for research which will aim to quantify and identify predictive patterns between financial hardship, mental health, and help-seeking in single parents. Follow on studies exploring the issues raised by this paper in other contexts should also consider variables such as the income level of single parents and whether single parents with higher income are less impacted by psychological distress. Furthermore, more consideration needs to be given to the geographical location of single parents, for example, studies have highlighted the difficulties faced by parents in rural locations verses inner city locations (Simmons et al. 2007). Due to the qualitative nature of the present study these factors were not taken in to account, but our future quantitative research will give include an analysis of these variables. A further limitation of this study was that recruitment was based on a self-selecting opportunity based approach. However, this resulted in a sample dominated by female participants. This was not unexpected as 90% of single parents are female (Great Britain. Office for National Statistics 2016). Furthermore, single mothers have been found to have significantly less income than single fathers (Hilton and Kopera-Frye 2006), therefore, may experience the stressors associated with financial hardship and parenting to a greater extent. However, we acknowledge that some of the emerging themes may be constructed with differing narratives if the experiences of single fathers were explored.

Past research has identified that single parents in the United Kingdom face significant financial hardships. This study has highlighted that the stresses of parenting alone appear to heighten feelings of stress, uncertainty, and depression associated with finances. Therefore, it is vital that health and mental health services recognise this distress, and understand the impact that financial difficulties have upon single parents. It is important to consider the sources of help available to single parents particularly help related to mental health and help focused on helping long parents cope with financial hardship. A focus on mental health support for single parents in need may have an additional impact upon the adjustment and wellbeing of children growing up in single parent households.
